# Clinical and Biological Manifestation of RNF168 Deficiency in Two Polish Siblings

**DOI:** 10.3389/fimmu.2017.01683

**Published:** 2017-12-04

**Authors:** Barbara Pietrucha, Edyta Heropolitańska-Pliszka, Robert Geffers, Julia Enßen, Britta Wieland, Natalia Valerijevna Bogdanova, Thilo Dörk

**Affiliations:** ^1^Department of Immunology, Children’s Memorial Health Institute, Warsaw, Poland; ^2^Genome Analytics Unit, Helmholtz Center for Infection Research, Braunschweig, Germany; ^3^Gynaecology Research Unit, Hannover Medical School, Hannover, Germany; ^4^Radiation Oncology Research Unit, Hannover Medical School, Hannover, Germany

**Keywords:** DNA repair, chromosome instability, radiosensitivity, immunodeficiency syndrome, double-strand break repair

## Abstract

Germline mutations in the RING finger protein gene *RNF168* have been identified in a combined immunodeficiency disorder called RIDDLE syndrome. Since only two patients have been described with somewhat different phenotypes, there is need to identify further patients. Here, we report on two Polish siblings with RNF168 deficiency due to homozygosity for a novel frameshift mutation, c.295delG, that was identified through exome sequencing. Both patients presented with immunoglobulin deficiency, telangiectasia, cellular radiosensitivity, and increased alpha-fetoprotein (AFP) levels. The younger sibling had a more pronounced neurological and morphological phenotype, and she also carried an *ATM* gene mutation in the heterozygous state. Immunoblot analyses showed absence of RNF168 protein, whereas ATM levels and function were proficient in lymphoblastoid cells from both patients. Consistent with the absence of RNF168 protein, 53BP1 recruitment to DNA double-strand breaks (DSBs) after irradiation was undetectable in lymphoblasts or primary fibroblasts from either of the two patients. γH2AX foci accumulated normally but they disappeared with significant delay, indicating a severe defect in DSB repair. A comparison with the two previously identified patients indicates immunoglobulin deficiency, cellular radiosensitivity, and increased AFP levels as hallmarks of RNF168 deficiency. The variability in its clinical expression despite similar cellular phenotypes suggests that some manifestations of RNF168 deficiency may be modified by additional genetic or epidemiological factors.

## Introduction

Primary immunodeficiency syndromes commonly associate with functional impairments in DNA double-strand break (DSB) repair, highlighting the critical nature of this pathway for the development and maturation of the immune system (1, 2). A genetic disorder including “radiosensitivity, immunodeficiency, dysmorphic features, and learning difficulties,” coined RIDDLE syndrome, has initially been described in a single patient whose cells lacked the ability to recruit 53BP1 to DSB sites (3). Subsequent work revealed that the *RNF168* gene was mutated in this patient (4).

*RNF168* encodes an E3 ubiquitin ligase that orchestrates the accumulation of 53BP1 or BRCA1 to DNA lesions (4, 5). RNF168 binds to ubiquitinated linker histone H1 at sites of DNA damage (6) where it then ubiquitinates histones H2A and H2AX, a prerequisite for accurate DSB repair (5, 7–10). RNF168-dependent ubiquitination of histones generates docking sites for RAP80 that mediates binding of BRCA1 to sites flanking the break (11) and also generates one of the histone marks required for 53BP1 recruitment (12, 13). RNF168 has been reported to further promote the initial recruitment of 53BP1 through its direct polyubiquitination (14). 53BP1 in turn suppresses end resection and promotes non-homologous end-joining in antagonism with BRCA1 (15). Consistent with its role in 53BP1 activation, RNF168 is required for class switch recombination and for V(D)J recombination (16, 17).

The second patient with RNF168 deficiency has been described in the literature with a somewhat different clinical phenotype including mild gait ataxia, ocular telangiectasia, elevated AFP, immunodeficiency, microcephaly, growth retardation, and terminal respiratory failure (18). Again, patient cells lacked RNF168 expression and the ability to recruit 53BP1 to sites of DNA DSBs. By contrast with the originally described patient, the clinical description of this patient indicated normal intelligence but a phenotype resembling ataxia–telangiectasia (18).

Given the rarity and non-overlapping features of the syndrome, it will be important to identify additional patients, describe consistent clinical phenotypes or differences, and better define the clinical spectrum of this disorder. In this work, we report on two Polish siblings with RNF168 deficiency.

## Patients and Methods

### Case Reports

The older boy, now aged 21 years, was born at term with asphyxia, hypotrophy (2,100 g), and second-degree intraventricular hemorrhage. He was rehabilitated until 2 years of age. He started to walk alone at the age of 1.5 years, then slightly abnormal gait and some “motor clumsiness” were observed. Since the neonatal period he was suffering from recurrent respiratory infection including pneumonia, a few events of bronchitis and chronic sinusitis. At the age of 7 years, he was hospitalized due to suspicion of seizure disorders, to be differentiated with tics. He was found to have reduced level of eye–hand coordination and elevated levels of emotional tension. The severity of health problems increased by the age of 13, when he was again hospitalized several times because of recurrent respiratory infections, sinusitis, and nocturnal enuresis. By age 14, he was hospitalized because of recurrent fever, generalized chronic lymphadenopathy, gait disturbances, mild arthritis, and erythematous, scaly skin lesions on lower limbs. Based on biopsies of lymph nodes and bone marrow and imaging tests, a proliferative process was excluded. Although the skin lesions clinically resembled those of vascular origin, a pathological examination of skin biopsy did not reveal any signs of vasculitis. Due to an increased level of serum AFP [59.1 IU/mL (normal range < 5 IU/mL)], cancer of testis was excluded. MRI of brain showed lesions localized in white matter to be differentiated with inflammation, vasculitis, or demyelination. EMG revealed slight chronic neurogenic, axonal damage of muscles of the lower limbs, suggesting demyelinating component or sensomotor neuropathy. Finally, the only diagnosis the boy was given was mononucleosis but since then he has been suffering from repeated headaches. By the age of 15, he was admitted to the Department of Immunology due to hypogammaglobulinemia. His total IgG was 4.29 g/L (range for age 7.06–14.40 g/L) and his IgA was below 0.06 g/L (range for age 0.85–1.94 g/L). He presented with conjunctival telangiectasia, persistent skin lesion on his lower limbs, chronic sinusitis, recurrent headaches, and stomach pains. His karyotype was 46,XY, but a few translocations were found involving chromosomes 7q and 14. Based on laboratory tests, we suspected ataxia–telangiectasia and regular substitution of intravenous immunoglobulin was commenced. By the age of 16, the boy was again hospitalized due to abdominal pain, prolonged diarrhea, and significant weight loss. Gastroscopy and colonoscopy were performed and *Helicobacter pylori* infection was confirmed but inflammatory bowel disease was excluded.

His younger sister, now aged 12 years, was born at term after an uneventful pregnancy and suffered from her first infection of upper respiratory tract by the age of 6 months. Since early childhood she presented with psycho-motor developmental delay. By the age of 6 years, she was consulted in the Immunology Outpatient Clinic Children’s Memorial Health Institute (CMHI) due to recurrent mucosal herpes simplex infections. Up to now she has had a few upper respiratory tract infections including bronchitis and otitis, and for 9 years an increasing number of herpes infections. She had a decent value for total IgG of 8.85 g/L (normal range for age 8.53–14.40 g/L) but reduced IgA < 0.06 g/L (normal range for age 0.38–2.35 g/L). IgG subclass analysis revealed reduced IgG2 at 0.39 g/L (normal range for age 0.71–3.41 g/L). Like her brother, she had markedly increased serum AFP at 41.9 IU/mL (normal range < 5 IU/mL).

At the time of this manuscript, there was no evidence of growth failure in both siblings. The brother’s weight was 105.5 kg (3.69 SD, >97c), height was 182.5 cm, head circumference 59.5 cm (1.7 SD), and BMI 31.68 (3.73 SD). The sister’s weight was 66 kg (3.04 SD, >97c), height 160.5 (1.56 SD, 90c), head circumference 52 cm (−1.37 SD, 10c), and BMI 25.54 (2.57 SD, >97c). Written informed consent was obtained from the patients for the publication of these case reports and their photographs (Figure S1 in Supplementary Material).

### Cell Culture

Lymphoblastoid cell lines (LCLs) were established using routine EBV immortalization of B-lymphocytes from peripheral EDTA blood samples for each of both patients and were grown in RPMI 1640 supplemented with 10% fetal calf serum, 500 U/mL penicillin, 0.5 mg/mL streptomycin, and 2 mM l-glutamine. The LCLs were designated HA591 for the sister and HA592 for her older brother. Previously established LCLs from a healthy donor (HA325) and from a patient with classical ataxia–telangiectasia (HA56) were maintained under the same cell culture conditions. Primary fibroblast cultures were obtained from skin biopsies for each of both patients and were grown in DMEM with the above-mentioned supplements. The fibroblast lines were designated F591 for the sister and F592 for her older brother. The previously described ADP fibroblast line from a healthy male donor was cultured under the same conditions (19). All cell cultures were maintained at 37°C in a humidified atmosphere with 5% CO_2_. For treatment with ionizing radiation, cells were γ-irradiated at the respective doses (1.5 and 6 Gy) using a Mevatron MD-2 accelerator (Siemens, Munich, Germany).

### Genetic Analysis

Genomic DNA was extracted from peripheral blood lymphocytes or cultured lymphoblastoid cell lines using proteinase K digestion and phenol–chloroform extraction. Exome sequencing was performed on genomic DNA samples (1 µg) from each of both affected siblings. For this purpose, exonic sequences were enriched using the SureSelect XT Human All Exon V6 library (Agilent Technologies, Santa Clara, CA, USA) and were analysed on an Illumina HiSeq2500 platform using TruSeq SBS Kit v3-HS (200 cycles, paired end run) with an average of 25 × 10^6^ reads per single exome and 100× coverage (Illumina Inc., San Diego, CA, USA). Raw exome sequencing data were called, de-multiplexed, and aligned according to the GATK pipeline and variants were annotated using the SnpEff tool (http://snpeff.sourceforge.net/SnpEff.html). Mutations were filtered according to their minor allele frequencies in the NCBI SNP and/or 1000Genomes databases and according to their predicted effects. The truncating mutation in *RNF168* was then confirmed by Sanger sequencing using BigDye chemistry and a Genetic Analyzer 3100 Avant (Applied Biosystems, Foster City, CA, USA). Primers for validation sequencing of the RNF168*c.295delG mutation were 5′-GGACAAAATCTTGCCCTTGAC-3′ and 5′-ACCCGAAGAAATTCTCTCGTC-3′. Primers for the sequencing of the ATM*1402_1403delAA mutation were 5′-CTATGGAAATGATGGTGATTCTC-3′ and 5′-GCATCTGAAATAGAATTTGACATC-3′ (20). Purified PCR products were subjected to direct sequencing using BigDye v1.1 terminator chemistry and a 3100 Avant capillary sequencer (Life Technologies). Sequencing data were analyzed with the Sequencing Analysis 5.1.1 software. Patient and parental genomic DNAs were further genotyped by means of RFLP analysis. For this assay, 10 µL genomic PCR products were generated with the *RNF168* primer pair listed above and were incubated overnight with 1.5 U *Mnl*I (New England Biolabs). Cleavage fragments were separated through electrophoresis on 2% agarose gels supplemented with GelRed and were visualized over an UV transilluminator.

### Immunoblotting

Cells were lysed in cell extraction buffer (50 mM Tris pH 7.4, 150 mM NaCl, 2 mM EGTA, 2 mM EDTA, 25 mM NaF, 0.1 mM Na_3_VO_4_, 0.1 mM PMSF, 2 mg/mL Leupeptin, 2 mg/mL Aprotinin, 0.2% Triton X-100, 0.3% NonidetP-40) for 30 min on ice and centrifuged at 16100 rcf for 15 min. Protein extracts were separated through SDS-PAGE followed by immunoblotting. Primary antibodies against the following proteins were used: ATM (rabbit monoclonal, Epitomics, 1:1,000), DNA-PKcs (mouse monoclonal, Calbiochem, 1:500), pSer824-KAP1 (rabbit monoclonal, Bethyl Laboratories, 1:5,000), RNF168 (rabbit polyclonal, GeneTex, 1:1,000), and β-Actin (mouse monoclonal, Sigma, 1:3,000). Anti-mouse and anti-rabbit horseradish peroxidases labeled secondary antibodies were purchased from GE Healthcare. Enhanced chemiluminescence (Dura ECL, Thermo Scientific/Pierce) was used for visualization of immunoreactive bands.

### Immunocytochemistry

Subconfluent fibroblast cells grown on cover glasses in six-well plates or lymphoblastoid cells centrifuged onto slides through cytospin were fixed with 3% (w/v) PFA, 2% (w/v) sucrose for 10 min. Cells were permeabilized with 0.2% (v/v) Triton X-100 in 1× PBS for 3 min and rinsed three times with 1× PBS. Cells were incubated for 1–1.5 h at room temperature with the primary antibodies against Histone H2A.x Phospho(S139) (Millipore, 1:200) and 53BP1 (Bethyl Laboratories, 1:200) in 2% (w/v) normal goat serum (Dianova). After 1× PBS washing, cells were incubated with Alexa Fluor anti-mouse IgG 488 and Alexa Fluor anti-rabbit IgG 546 (Invitrogen) for 45 min to 1 h at room temperature in the dark. Cells were washed with 1× PBS, and DNA was counterstained with 4′,6-diamidino-2-phenylindole (DAPI) (Invitrogen) (1:50,000 in PBS) for 5 min and mounted using Prolong Gold^®^ (Invitrogen). Foci were counted under a Leica DMI 6000B fluorescence microscope, and cells with *n* > 4 foci were considered “positive” cells. Results from *n* = 100–150 cells with two technical replicates of each line were analyzed. Mean values of RNF168-deficient cells and RNF168-proficient reference lines were compared with two-sample *t*-tests. Accounting for multiple testing, *p*-values were considered significant at α < 0.01.

## Results

Two Polish siblings presented with a primary immunodeficiency of unknown cause at the Department of Immunology, The CMHI, Warsaw (21). They had been born to healthy consanguineous parents (third degree cousins). Pictures at their present age are provided as Figure S1 in Supplementary Material, and extended case reports are provided in the Section “Patents and Methods.” Both siblings showed normal growth and no signs of microcephaly or ataxia, no café-au-lait spots or gray hair, although the younger sister has some mild facial dysmorphism and showed clumsy gait at earlier childhood. Conjunctival telangiectasia was noted in both siblings. Mild mental retardation with learning difficulties was apparent for the sister but not for her older brother, both siblings still suffer from nocturnal enuresis. Laboratory measurements revealed IgA and IgG2 deficiency in the sister, while the brother had markedly reduced total IgG which required starting regular IVIG, later SCIG substitution in 2010. While lymphocyte subpopulations were within the normal range for their age, reduced percentage, and number of memory B cells and “switched” B cells were found in both siblings. Both siblings also had strongly elevated serum levels of AFP. Cellular radiosensitivity, as measured by means of colony survival assays in lymphoblastoid cells, was markedly increased for both siblings.

**Figure 1:**
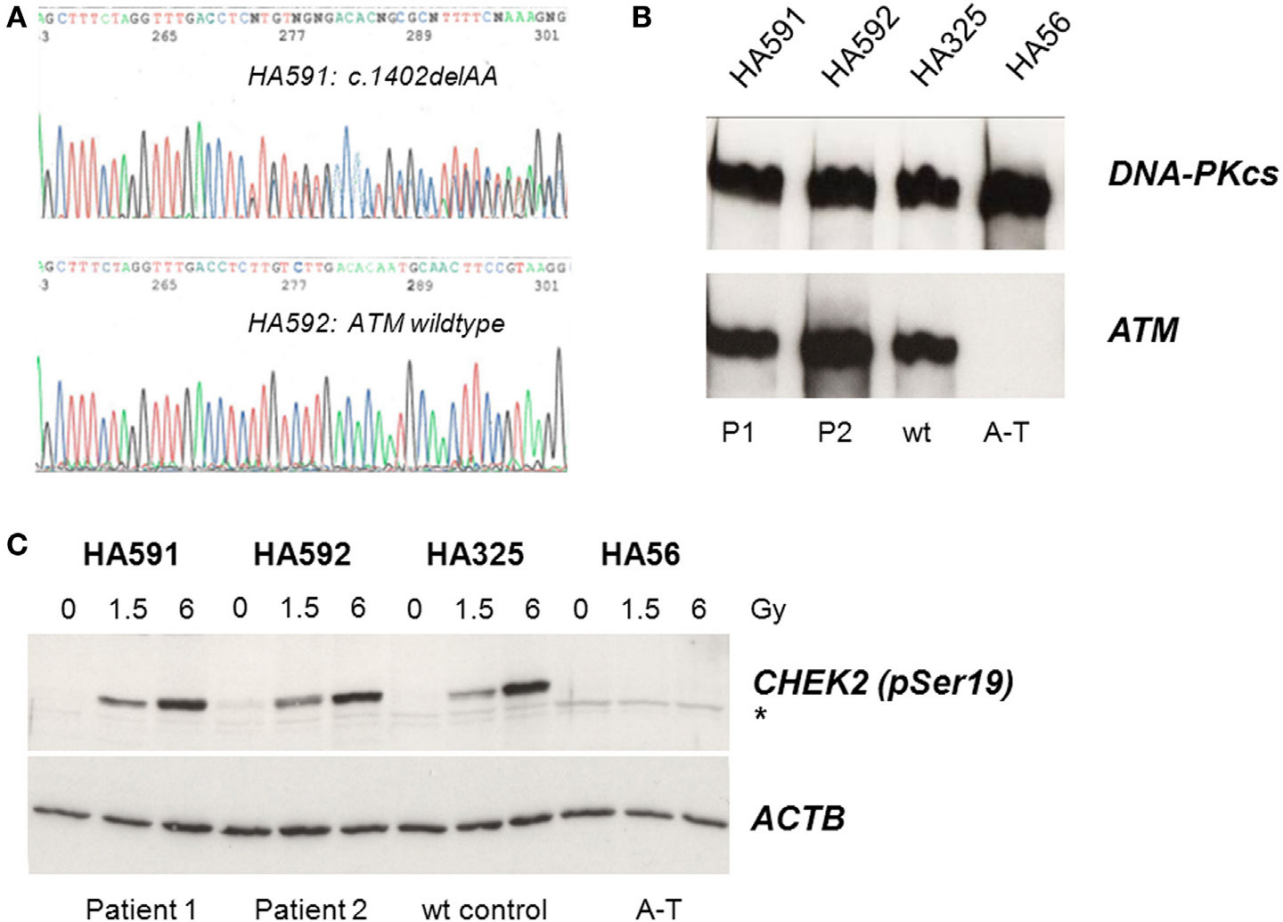
Assessment of ATM mutation **(A)**, ATM protein level **(B)**, and ATM kinase activity **(C)**. **(A)** Direct sequencing of *ATM* exon 12 reveals heterozygosity for the novel frameshift mutation c.1402_1403delAA in genomic DNA from the sister (HA591) but not the brother (HA592). **(B)** Immunoblotting detects ATM protein in lymphoblastoid cells from both patients (HA591, HA592). Lymphoblastoid cell lines (LCLs) from a healthy individual were used as an ATM-proficient control (HA325), and LCLs from a patient with classical ataxia–telangiectasia were used as an ATM-deficient control (HA56). DNA-dependent protein kinase, catalytic subunit, served as the loading control (DNA-PKcs). **(C)** Immunoblotting of cells after irradiation reveals radiation-induced phosphorylation of the ATM substrate KAP1 at Ser824. Cells were irradiated with 0, 1.5, or 6 Gy, respectively, and proteins were extracted at 30 min after irradiation. LCLs from a healthy individual were used as an ATM-proficient control (HA325), and LCLs from a patient with classical ataxia–telangiectasia were used as an ATM-deficient control (HA56). β-actin served as the loading control (ACTB).

Because of the IgG and IgA deficiency, cellular radiosensitivity, telangiectasia, and elevated AFP, we explored the possibility of an ATM-related dysfunction. Although the siblings showed no ataxia, it is known that very mild form of ataxia–telangiectasia exist where the cerebellar phenotype is attenuated and occurs late in life (22–24). Indeed, direct sequencing of germline DNA demonstrated a novel *ATM* gene mutation, c.1402_1403delAA, in one of the two siblings (Figure 1A). However, this mutation was carried by the sister only in the heterozygous state and was not shared with her brother. Subsequent immunoblot analyses of protein extracts obtained from lymphoblastoid cells revealed wild-type levels of ATM protein in the cell lines from both siblings (Figure 1B). To further investigate the functional proficiency of the ATM pathway, we irradiated lymphoblastoid cells from both siblings at 1.5 or 6 Gy and monitored the phosphorylation of KAP1 (Ser824), a known target of the ATM kinase (25). We did not observe any significant difference between the patient cells and a wild-type control, demonstrating largely normal ATM kinase activity (Figure 1C). Thus, the heterozygosity for an *ATM* mutation in one of the two patients was insufficient to explain their clinical phenotype.

**Figure 2:**
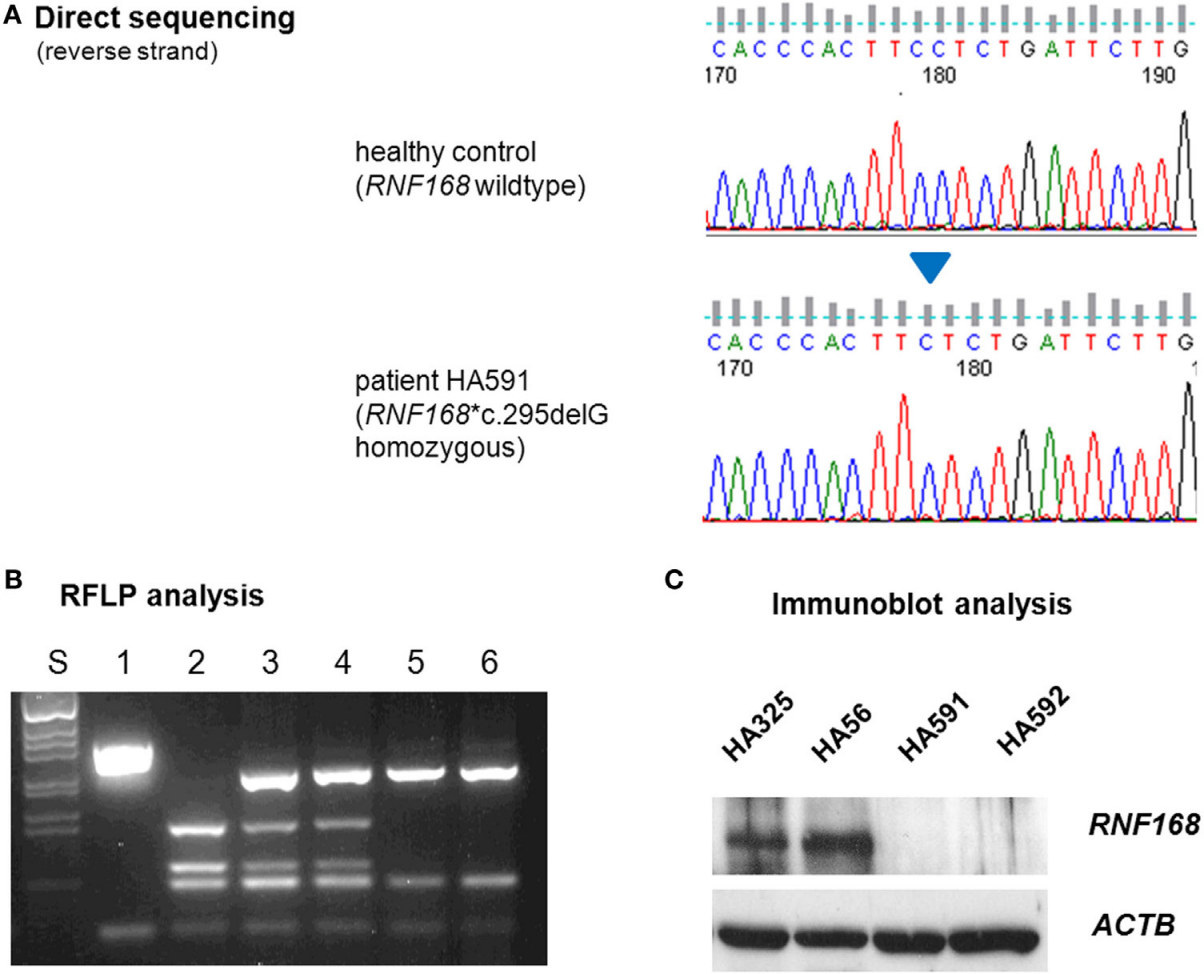
Assessment of RNF168 mutation **(A)** and RNF168 protein level **(B)**. **(A)** Direct sequencing of *RNF168* exon 12 reveals homozygosity for the novel frameshift mutation c.295delG in genomic DNA from either of both patients (HA591, HA592). **(B)** RFLP analysis of PCR products on 2% agarose gel electrophoresis. S, size marker; 1, undigested PCR product; 2–6, PCR products cleaved with *Mnl*I: 2, wild-type control, 3, paternal sample, 4. maternal sample, 5, patient HA591, 6, patient HA592. **(C)** Western blot analysis reveals strongly reduced immunoreactivity for RNF168 protein in lymphoblastoid cells from either of both patients (HA591, HA592). Lymphoblastoid cell lines (LCLs) from a healthy individual were used as an RNF168-proficient control (HA325), and LCLs from a patient with classical ataxia–telangiectasia were used for comparison (HA56). β-actin served as the loading control (ACTB).

To elucidate the molecular basis for the immunodeficiency, we performed exome sequencing on genomic DNA samples from both patient LCLs. We identified one novel truncating mutation in *RNF168*, c.295delG, that appeared in the homozygous state (Figure 2A) and was shared by both siblings. Conceptual translation with this mutation results in a frameshift that starts at codon 99 and generates a premature termination signal 17 codons downstream (p.Glu99Lysfs*17). Because *RNF168* mutations had previously been associated with an immunodeficiency (RIDDLE) syndrome in two unrelated patients (4, 18), this mutation was considered for further analyses. Genomic DNA samples from both parents were genotyped using RFLP analysis and direct sequencing and were confirmed to be heterozygous, thereby proving true homozygosity of the affected patients (Figure 2B). We then assessed the amount of RNF168 protein using immunoblot analyses of lymphoblastoid cells from both patients. By contrast with wild-type control and with ataxia–telangiectasia cells, we could not detect RNF168 protein in the LCLs from both patients (Figure 2C).

**Figure 3:**
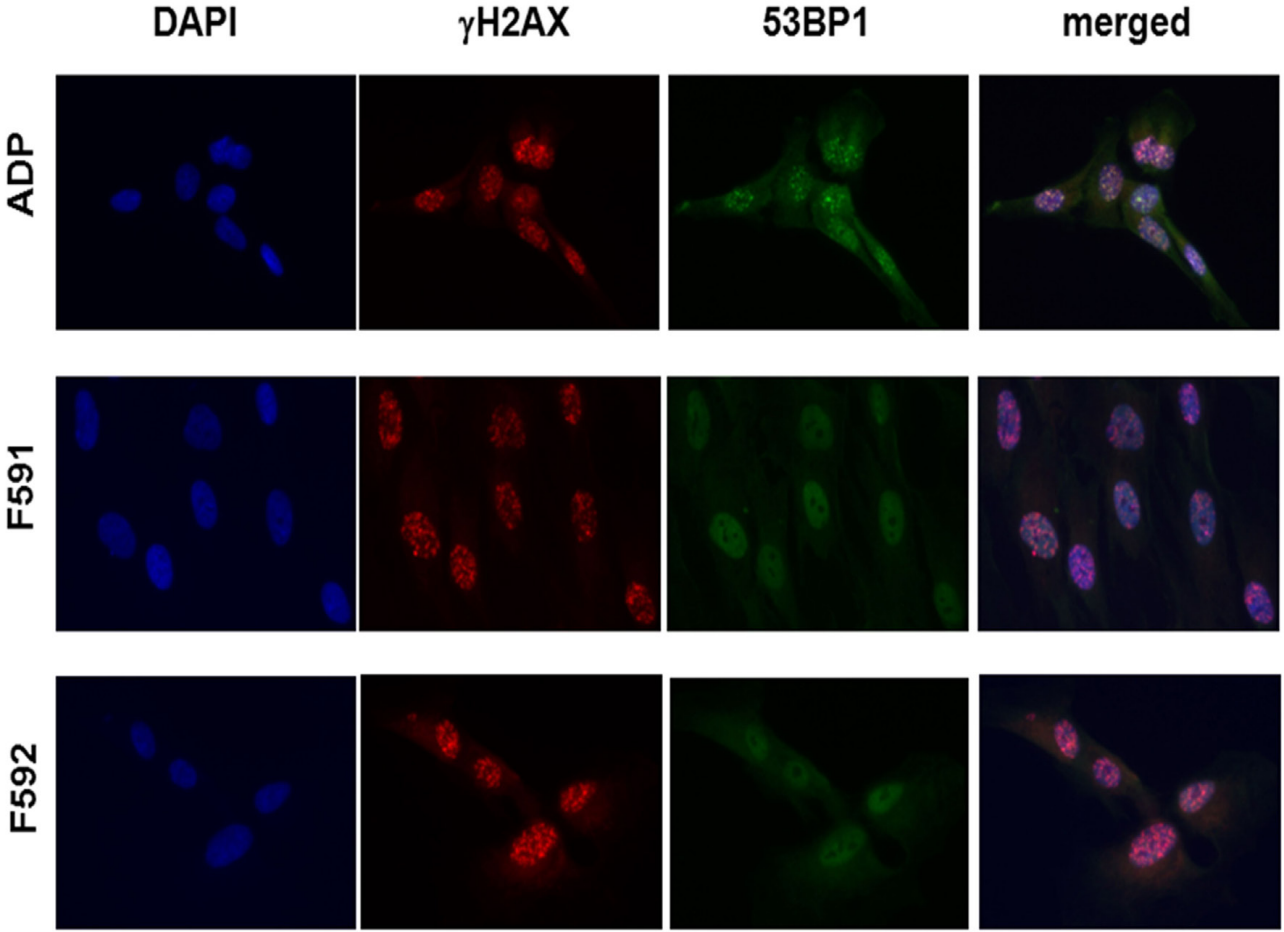
Immunocytochemical analysis of irradiation-induced repair foci in RNF168-deficient fibroblasts and lymphoblastoid cell lines. Detection of γH2AX foci (second column) and 53BP1 foci (third column) in reference ADP fibroblasts (upper panel) compared to patient fibroblasts F591 and F592 (middle and bottom panel) at 1 h after 6 Gy irradiation. DAPI staining and merged pictures are shown in the outer columns as controls for intracellular localization.

Because the patient cells had shown cellular radiosensitivity in the initial assay and RIDDLE cells had been reported to be defective in 53BP1 recruitment (3), we comparatively monitored the formation and disappearance of γH2AX and 53BP1 repair foci over time in both, lymphoblastoid cells and fibroblasts from both patients. Whereas the initial formation of γH2AX occurred near normally at 1 h after irradiation with 6 Gy, the fibroblasts from both patients failed to form any detectable 53BP1 foci (Figure 3; Table 1). Similarly, we could not detect any 53BP1 foci after irradiation in lymphoblastoid cells from both patients (data not shown). At later time-points of 48 or 72 h post-irradiation, we observed significantly more residual γH2AX foci in the patient cells than in wild-type control lymphoblasts or fibroblasts (*p* ≤ 0.002), consistent with a pronounced defect in the repair of DNA DSB damage. At 72 h, some 38–44% of RNF168-deficient fibroblasts were still γH2AX-positive compared with 1–3% of reference fibroblasts (Table 1).

**Table 1:** Monitoring DNA repair protein foci in fibroblasts with RNF168 deficiency.

Fibroblast line	Mean foci per cell			Percentage of foci-positive cells		

	53BP1	γH2AX	p_(**γ**H2AX)_	53BP1	γH2AX	p_(**γ**H2AX)_^a^
**ADP reference**						
1 h after 6 Gy	11 ± 3	43 ± 5	Ref	28 ± 1	67 ± 1	Ref
24 h after 6 Gy	3 ± 0	6 ± 2	Ref	18 ± 1	39 ± 8	Ref
48 h after 6 Gy	2 ± 0	3 ± 4	Ref	9 ± 1	13 ± 1	Ref
72 h after 6 Gy	1 ± 0	2 ± 2	Ref	7 ± 1	2 ± 1	Ref
**F591 RNF168 deficient**						
1 h after 6 Gy	0	44 ± 1	0.80	0	71 ± 0	0.01
24 h after 6 Gy	0	13 ± 2	0.08	0	58 ± 1	0.07
48 h after 6 Gy	0	11 ± 3	0.12	0	45 ± 2	**0.002**
72 h after 6 Gy	0	9 ± 1	0.05	0	39 ± 1	**0.0009**
**F592 RNF168 deficient**						
1 h after 6 Gy	0	52 ± 1	0.13	0	71 ± 2	0.12
24 h after 6 Gy	0	12 ± 1	0.06	0	61 ± 1	0.06
48 h after 6 Gy	0	11 ± 3	0.12	0	59 ± 2	**0.001**
72 h after 6 Gy	0	9 ± 0	0.04	0	43 ± 1	**0.0007**

*The number of 53BP1 or γH2AX foci per cell and the percentage of foci-positive cells were assessed in primary fibroblasts from the two patients with RIDDLE syndrome (F591, F592) and wild-type fibroblasts from a healthy donor (ADP) at five time-points (0, 1, 24, 48, 72 h). Shown are the mean values from two technical replicates after normalization to the untreated condition (*t* = 0). Because no 53BP1 foci were detected in F591 and F592, the monitoring over time was only performed for γH2AX foci and *p*-values were determined by two-sample *t*-tests using ADP as the reference*.

*^a^p < 0.01 marked in bold*.

## Discussion

Patients suspected of immunodeficiency frequently have underlying DNA DSB repair defects with considerable impact on V(D)J recombination, class switching and lymphocyte maturation, leading to increased infections and cancer risk (1, 2). In addition, the phenotype of cellular radiosensitivity may identify immunologically compromised patients with increased toxicity to radiation and chemotherapeutic agents. Because such disorders are often partial phenocopies of one another, they have been tentatively classified as a “XCIND” group of disorders characterized by X-ray sensitivity, cancer susceptibility, immunodeficiency, neurological abnormalities, and DNA DSB repair dysfunction (1). Modulation and sensing of damage-induced histone ubiquitylation strongly impact on DSB repair pathway choice and genome integrity, as well as cell and organismal fitness, and RNF168 plays a unique role in being a writer as well as a reader of the histone ubiquitylation code (15). In this work, we have identified two siblings with RNF168 deficiency, also termed RIDDLE syndrome, for which only two single cases have been published previously (3, 18). Our genetic testing results for two homozygous siblings and their unaffected heterozygous parents corroborate the autosomal recessive inheritance of this disorder. As a clinical phenotype may be influenced by many genetic and epidemiological factors, it is important to collect clinical data from several patients of a rare syndrome to identify a common theme emerging from a defined molecular deficiency.

The first patient reported had marked IgG deficiency, mild motor control and learning difficulty, mild facial dysmorphism, and short stature (3). However, the second reported patient featured ataxia, telangiectasia, elevated AFP, IgA deficiency, microcephaly, and pulmonary failure (18). In this study, even though one of the two siblings also was heterozygous for an A–T mutation, the RNF168 deficiency resulted in a much milder neurological phenotype, if any, with the major clinical feature being the immunological defect. The lack of classic ataxia was in line with the ATM proficiency measured *in vitro* in both patients’ lymphoblastoid cell lines. Table 2 compares the features reported for all four hitherto described patients with RNF168 deficiency.

**Table 2:** Comparison of diagnostic features of reported patients with RNF168 deficiency.

	Patient 1 (15-9BI) (3, 4)	Patient 2 (RS66) (18)	Patient 3 (HA591) (this study)	Patient 4 (HA592) (this study)
Country of origin	UK	Turkey	Poland	Poland
Sex	Male	Male	Female	Male
Age	22	16	13	21
*RNF168* mutation (cDNA level)	c.397dupG/c.1323_1326delACAA	c.391C > T homozygous	c.295delG homozygous	c.295delG homozygous
*RNF168* mutation (protein level)	p.A133Gfs*10/p. N441Rfs*16	p.R131X homozygous	p.E99Kfs*17 homozygous	p.E99Kfs*17 homozygous
*ATM* mutation	No	No	c.1402_1403delAA heterozygous	No
Cellular radiosensitivity	Yes	Yes	Yes	Yes
Reduced IgG	Yes	No	Yes (IgG2)	Yes
Reduced IgA	(No)^a^	Yes	Yes	Yes
Elevated AFP	n.d.	Yes	Yes	Yes
Ataxia	(No)^b^	Yes, mild	(No)^b^	(No)^b^
Telangiectasia	No	Yes	Yes	Yes
Short stature	Yes	Yes	No	No
Microcephaly	(No)^c^	Yes	(No)^c^	No
Learning difficulties	Yes	No	Yes	No
Respiratory failure	No	Yes	No	No

*Clinical and cellular features were summarized for the first (15-9BI) and second (RS66) reported patients with RNF168 deficiency according to the descriptions by Stewart et al. (3) and Devgan et al. (18), and for the two new patients in the present report*.

*^a^Borderline in early childhood*.

*^b^Mild motor control difficulties*.

*^c^Mild facial dysmorphism*.

*Ig, immunoglobulin; AFP, alpha-fetoprotein*.

From this comparison, it becomes evident that RNF168 deficiency is consistently associated with immunoglobulin deficiency and cellular radiosensitivity, whereas ataxia, microcephaly, short stature, or learning difficulties were more variable phenotypes and not found in each of the four patients (Table 2). It is unlikely that the type of mutation in *RNF168* can explain the variability as all four patients harbor a truncation upstream of the WD40 domain that is crucial for homology-directed repair (26) and showed absence of RNF168 protein *in vitro*. It is possible that mutation in additional genes, including the *ATM* heterozygosity identified in the younger sister, may contribute as modifiers to phenotypic expressions. Interestingly, elevated serum AFP appears to be a common feature and may be of diagnostic use in future screening. We have further confirmed, in patient fibroblasts and in patient lymphoblast cells, a failure to form radiation-induced 53BP1 foci while γH2AX foci remain unaffected (3). Since there is no other human disorder known to show this phenotype, this may provide a rapid and specific diagnostic tool to distinguish RNF168 deficiency from phenocopies.

RNF168 deficiency is presently classified as combined immunodeficiency with syndromic features (27). Our study of two new patients refines reduced immunoglobulin levels and radiosensitivity as the hallmarks of this syndrome, confirms its unique cellular phenotype in the DNA damage response, and suggests elevated serum AFP as a useful laboratory marker. *RNF168* has occasionally been included on sequencing panels for primary immunodeficiencies (28, 29), and in one study two unspecified *RNF168* missense variants were reported in 1/33 patients (28). Another missense variant in *RNF168* has been observed in an adult patient with ZAP70 deficiency and was suggested to modify his phenotype (30). It is anticipated that, with the increasing use of next-generation sequencing in the diagnosis of immunodeficiency disorders (31), additional patients with *RNF168* mutations might be identified and can help to define and perhaps extend the clinical spectrum of RNF168 deficiency.

## Ethics Statement

This study was carried out in accordance with the Declaration of Helsinki. All subjects gave written informed consent, and the protocol was approved by the Bioethical Commission, The Children’s Memorial Health Institute, Warsaw.

## Author Contributions

BP and EH-P: clinical diagnosis and documentation; sample acquisition. RG, JE, BW, and NB: laboratory analyses. TD: data analyses and manuscript drafting. All authors: manuscript writing and approval.

## Conflict of Interest Statement

The authors declare that the research was conducted in the absence of any commercial or financial relationships that could be construed as a potential conflict of interest.

## Supplementary Material

Figure S1 Photographs of the Polish siblings with RNF168 deficiency at their age of 12 years (sister) and 21 years (brother).
