# Supplementary figures and images for: Clinical and Biological Manifestation of RNF168 Deficiency in Two Polish Siblings
Source: Front Immunol. 2017 Dec 4;8:1683. doi: 10.3389/fimmu.2017.01683 (PMC5722808; doi:10.3389/fimmu.2017.01683)

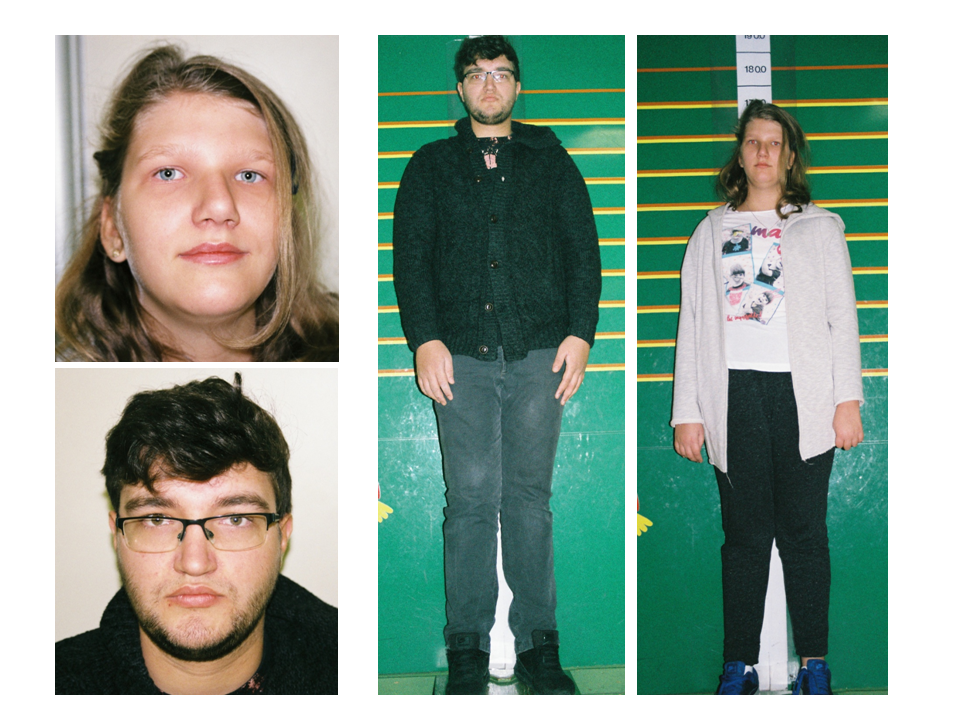

Supplement: Figure S1 — Photographs of the Polish siblings with RNF168 deficiency at their age of 12 years (sister) and 21 years (brother). [file Image_1.tif]
